# Gut microbiota alterations in golden snub-nosed monkeys during food shortage and parturition-nursing periods

**DOI:** 10.3389/fmicb.2025.1556648

**Published:** 2025-02-27

**Authors:** Guanwei Lan, Rui Ma, Yanshan Zhou, Zhantao Lu, Biqing Zhu, Juan Liu, Wei Wu, Yue Zhang, Jiabin Liu, Haijun Gu, Jie Lin, Wei Wei, Dunwu Qi

**Affiliations:** ^1^Sichuan Key Laboratory of Conservation Biology for Endangered Wildlife, Chengdu Research Base of Giant Panda Breeding, Chengdu, Sichuan, China; ^2^Key Laboratory of Southwest China Wildlife Resources Conservation (Ministry of Education), China West Normal University, Nanchong, China; ^3^College of Life and Environmental Sciences, Central South University of Forestry and Technology, Changsha, China; ^4^Administrative Bureau of Baihe National Nature Reserve, Aba, China; ^5^Sichuan Provincial Bureau of Forestry and Grassland, Chengdu, Sichuan, China

**Keywords:** golden snub-nosed monkeys, altruism, 16S rRNA, kin selection, evolutionary biology

## Abstract

Adopting unique survival strategies during spring food shortages and simultaneous parturition and nursing is crucial for golden snub-nosed monkeys. Social behaviors, such as altruism within one-male units (OMUs), are decisive for family health, but the role of microbiota in regulating these behaviors remains unknown. We conducted the gut microbiota from members of 10 OMUs using 16S RNA sequencing technology. We found that in adult males, gut microbiota diversity significantly decreased in food shortages and parturition-nursing period. Meanwhile, there was a notable reduction in 12 metabolism-related pathways, including those related to carbohydrates, amino acids, and lipid. The gut microbiota of adult male monkeys shifts from being enriched with the genera *Akkermansia* in winter to the genera *norank Muribaculaceae* in spring. This transition alters the pathways for nutrient acquisition, thereby reducing the consumption of stored energy. In contrast, other OMU members (adult females and subadults) did not experience adverse effects on the metabolic functions of their gut microbiota during the food-scarce spring, which is also a critical period for parturition and lactation in adult females. This study elucidates the co-evolution of altruistic behavior and gut microbiota in Sichuan snub-nosed monkeys, insights into the interaction mechanisms between mammalian microbiota and survival strategies.

## Introduction

Altruistic behavior in animals refers to actions taken by individuals to help other members of their species without direct personal gain (Daly, 1999; Samuni and Surbeck, 2023). This phenomenon is widely studied in biology and ethology, involving various theories and models (Seyfarth and Cheney, 1984). Altruistic behavior in animals is complex, encompassing multiple biological and psychological mechanisms (Kennedy et al., 2018; Samuni and Surbeck, 2023). Animal altruistic behaviors exhibit various manifestations, including cooperation and sharing (Huang et al., 2020; Schausberger et al., 2021). For instance, prairie dogs (*Cynomys* spp.) exhibited a form of cooperative altruistic behavior by emitting alarm calls when predators are detected (Kennedy et al., 2018). Interestingly, prairie dogs with kin present in their vicinity issue alarm calls significantly more frequently than those without kin, demonstrating altruistic behavior driven by kin selection (Kennedy et al., 2018).

The key concept within kin selection theory, which was proposed by Hamilton, suggested that altruistic behavior can evolve through natural selection if the reproductive benefits to the recipient, multiplied by the coefficient of relatedness, exceed the reproductive costs to the altruist (Bourke, 2014). The fundamental logic behind such altruistic behavior is that individual animals are more likely to help genetically related kin, thereby increasing the propagation of their own genes through the offspring of their relatives (West et al., 2002; Bourke, 2014). For instance, African wild dogs (*Lycaon pictus*) sacrifice individual interests during hunts to ensure group survival, enhancing overall group fitness (Fanshawe and Fitzgibbon, 1993). In cooperative breeding birds like long-tailed tits (*Aegithalos glaucogularis*), unsuccessful breeders may assist relatives in nest building, thereby contributing to the reproductive success of kin (Hatchwell et al., 2004). In primates, kin-biased altruism is particularly evident. For example, breeding individuals in large groups of black tufted-ear marmosets (*Callithrix kuhlii*) exhibit higher aggression toward strangers, potentially to maintain group size and prevent outsider intrusion, whereas those in smaller groups show more tolerance toward strangers, possibly to attract additional group members (French et al., 1995; Schaffner and French, 1997). Additionally, research on rhesus macaques (*Macaca mulatta*) indicates a preference for helping closer kin and engaging in mutual grooming with them (Butovskaya, 1993). These findings underscore the significance of kin selection in shaping altruistic behaviors in various animal species, highlighting the intricate balance between individual sacrifices and group benefits in the evolutionary landscape (Seyfarth and Cheney, 1984; Kennedy et al., 2018).

Golden snub-nosed monkeys (*Rhinopithecus roxellana*) are Asian colobines endemic to China living in a multilevel society, in which females reside in core social units consisting primarily of one male unit (OMU) with multiple females (Xiang et al., 2022). In this kind of social group, individual animals frequently exhibit altruistic behaviors based on kin selection. For example, some Yunnan snub-nosed monkeys (*Rhinopithecus bieti*) with more abundant milk actively nurse other infants within their family group to enhance the survival rate of the entire group’s offspring (Ren et al., 2012). Statistics show that during periods of food scarcity, over 87% of Sichuan snub-nosed monkey infants receive care from non-biological mothers (Xiang et al., 2019). This behavior not only alleviates the burden on other mothers but also provides the infants with additional nutrients and immune compounds, helping them to resist various parasites and diseases (Garnier et al., 2013; Pannaraj et al., 2017). Additionally, some non-pregnant female monkeys actively provide food and water to pregnant females, assisting them through the gestation period (Xiang et al., 2019). And pregnant females experience mobility challenges, other females also help care for their infants (Xiang et al., 2019). This series of altruistic behaviors not only enhances the survival chances of the pregnant females but also creates a better living environment for the soon-to-be-born infants (Clutton-Brock et al., 1989; Roulin, 2002). Unfortunately, no studies have yet demonstrated altruistic behavior by adult male Sichuan snub-nosed monkeys for the benefit of family development.

Altruistic behaviors that promote food sharing may interact with gut microbiota (Lewin-Epstein et al., 2017). For example, studies have found that in Sichuan snub-nosed monkey families, individuals with closer kinship, such as grandmothers and aunts, are more willing to provide milk to infants, which may lead to the transmission of gut microbiota (Lewin-Epstein et al., 2017; Liu et al., 2024). On the other hand, gut microbiota may regulate host behavior by influencing neurotransmitter levels, thereby indirectly promoting altruistic behavior (Bercik et al., 2011; Diaz Heijtz et al., 2011). Gut microbiota may spread and be preserved among family members through kin selection, while those detrimental to population development may be eliminated (Liu et al., 2024). Thus, the expression of altruistic behavior can influence the composition of gut microbiota.

As an endemic primate in China, golden snub-nosed monkeys are found in montane forests, where snow cover can last up to six months a year (Xiang et al., 2017). The prolonged winter and spring seasons pose significant challenges for the foraging and intake of food by golden snub-nosed monkeys, especially during the peak birthing period of pregnant females, which occurs around March each year (Xiang et al., 2017). When the increased nutritional demands of pregnancy and child-rearing coincide with the scarcity of food resources in winter and spring, the monkey population necessitates a brilliant survival strategy to maintain healthy population development. Based on this context, our study hypothesizes that adult male golden snub-nosed monkeys exhibit altruistic behavior by sharing food resources to ensure the food supply for pregnant and birthing females and to help juveniles survive the harsh winter and spring. This behavior is expected to be reflected in changes in their gut microbiota. To test this hypothesis, we conducted a year-long study tracking a band of Sichuan snub-nosed monkeys. We employed 16S rRNA high-throughput sequencing to explore the diversity and functional changes in the gut microbiota across different sex and ages throughout the seasons, inferring the expression levels of their altruistic behavior. The results of this study contribute to understanding the mechanisms linking altruistic behavior and gut microbiota in Sichuan snub-nosed monkeys, offering significant insights into their behavior and microbial characteristics. Besides, our conclusion will not only aid in the conservation of this rare primate but also provides new perspectives and methods for studying animal behavior and microbiology mechanisms.

## Materials and methods

### Study site

Our study was conducted in the Baihe National Nature Reserve in Sichuan. The Baihe National Nature Reserve (Latitude: 33°10′ - 33°22′ N; Longitude 104°01′ - 104°12′ E) is located in Aba Tibetan and Qiang Autonomous County, Sichuan Province. The Baihe National Nature Reserve is situated in the northern section of the Minshan Mountain range, with altitudes ranging from 1,240–4,453 meters, and features a temperate semi-humid climate (Kai Huang et al., 2021). The monkey groups are located in the Wangjiashan area (Latitude: 33°15′ N, Longitude: 104°08′ E, Altitude: 1,700–2,200 m), where the primary vegetation types include evergreen broadleaf forests, deciduous broadleaf forests, mixed coniferous-broadleaf forests, and alpine coniferous forests (Chu et al., 2018; Dong et al., 2019).

### Study subjects

All the subjects in our study were from the wild Sichuan snub-nosed monkey population in the Wangjiashan area of the Baihe Nature Reserve. Observation study began in May 2023. With the guidance of local conservation station staff, we spent five months acclimating the monkeys to our presence, allowing our researchers to observe the groups from a distance of 5–20 meters. Individual monkeys were identified using facial features (e.g., the shape of facial features and the presence of wrinkles), injury characteristics (e.g., scar shapes and physical disabilities), and hair traits. The population was determined to consist of 160 individuals, organized into 10 one-male units (OMUs) and an all-male unit (AMU). In all OMUs, the adult male-to-female ratio was 1:6.4. Each of the 10 OMUs included adults, subadults, juveniles, and infants.

### Diet

Following previous dietary research methods on golden snub-nosed monkeys, we conducted long-term tracking and observational plant identification (Liu et al., 2013). We found that their primary diet consists of various plants, including *Aralia chinensis*, *Acanthopanax evodiaefolius*, *Prunus brachypoda*, *Maddenia hypoleuca*, *Tilia chinensis*, *Tilia intonsa*, *Deutzia setchuenensis*, *Actinidia maloides*, *Elaeagnus angustata*, and *Sorbus tapashana*. Annually, their diet composition is: mature leaves 28.9%, young leaves 25.7%, fruits and seeds 19.1%, bark and stems 11.7%, with other items like flowers, buds, mosses, fungi, and bamboo shoots making up 14.7%.

### Food shortage and parturition-nursing period

Based on environmental changes in the natural habitat of Sichuan snub-nosed monkeys and their behavioral rhythms, the late food shortage period (November to April) coincides with the peak parturition period (mid to late March) and early nursing period (March to September), primarily in spring (February to April) (Xiang et al., 2017; Xiang et al., 2019). Thus, we focused on the diet during the food shortage and parturition-nursing period. During this time, Sichuan snub-nosed monkeys primarily consumed young leaves (32.6%) and bark (32.5%), with minor contributions from mature leaves (0.8%) and fruits/seeds (0.6%, Supplementary Table S1). This dietary composition continued the characteristics of winter food scarcity, dominated by bark, and contrasts significantly with their summer and autumn diets (Supplementary Table S1). In summer and autumn, their diet was consistent, with a higher proportion of mature leaves and lower bark consumption, and fruits/seeds accounted for 35.6%, highlighting the nutritional abundance in autumn (Supplementary Table S1). These seasonal dietary differences underscore the food scarcity during the food shortage and parturition-nursing period.

### Sample collection and transportation

This study focuses on the altruistic behavior exhibited by male Sichuan snub-nosed monkeys in OMUs in response to food scarcity. Therefore, all samples were collected from individuals within OMUs, and no samples were taken from AMUs. The collection of fecal samples was facilitated by individual identification through observation method and the monkeys’ acclimation to the researchers. Specifically, researchers quietly observed the monkey groups from a distance of 5–20 meters. Upon observing defecation, researchers promptly approached to collect the feces, ensuring that the time from defecation to collection was <20 min. Each individual’s fecal sample was collected only once per season, with efforts made to complete the collection for all individuals within the same month of each season.

Collection personnel strictly adhered to sample collection protocols, wearing disposable surgical masks and PE gloves. Once the monkeys defecated, the feces were transferred to disposable sterile surgical drapes. The outer layer of the feces was gently removed, and the internal portion was placed into 50 mL sterile cryogenic vials. After labeling, the samples were rapidly frozen in sampling transport boxes containing dry ice to ensure that the gut microbiota remained unaffected by external environmental factors. The samples were subsequently transferred to a laboratory and stored in a − 80°C ultra-low temperature freezer until DNA extraction. Using the methods described above, we collected a total of 194 fecal samples throughout the four seasons. Of these, 35 samples were from adult males (AM), 66 from adult females (AF), 45 from subadult males (SM), and 48 from subadult females (SF). All samples were grouped according to the age class and season of the donors. The specific numbers, season and groups of sample collection are detailed in Supplementary Table S2.

### DNA extraction and 16S rRNA gene sequencing

The collected fecal samples were pretreated using magnetic beads (Xue et al., 2015), and the total microbial DNA was extracted from the feces using the Qiagen QIAamp DNA Stool Mini Kit (Qiagen, Germany) and following the kit protocols for isolation of DNA. The DNA quality of each sample was determined by 1% agarose gel electrophoresis, and sent to Shanghai Sangon Biotech Technology Co., Ltd. (Shanghai, China) for further concentration, assessment, amplification, and sequencing according to their standard procedures (Wang et al., 2016; Yin et al., 2016). The V3–V4 regions of the 16S rRNA gene were amplified using the primers 338F (5’-ACTCCTACGGGAGGCAGCAG-3′) and 806R (5’-GGACTACGCGGGTATCTAAT-3′), which target conserved sequences found in bacteria. PCR reactions were performed in triplicate 20 μL mixtures containing 4 μL of 5× FastPfu Bufer, 2 μL of 2.5 mM dNTPs, 0.8 μL of each primer (5 μM), 0.4 μL of FastPfu Polymerase, and 10 ng of template DNA. The amplicons were then extracted from 2% agarose gels and further purified by using the AxyPrep DNA Gel Extraction Kit (Axygen Biosciences, Union City, CA, USA) and quantified by QuantiFluor-ST (Promega, USA) according to the protocols. Purified amplicons were pooled in equimolar and paired-end sequenced (2 × 300) on an Illumina MiSeq platform (Illumina, San Diego, USA) according to the instructions. The raw reads were deposited into the NCBI Sequence Read Archive (SRA) database (BioProject: PRJNA1172943).

### Statistical analysis

Raw reads were demultiplexed and quality-filtered using QIIME (version 1.9.1). The taxonomy of each 16S rRNA gene sequence was analyzed against the SILVA 128/16 s bacteria database with confidence threshold of 70%. After a smallest sequencing depth and clustering, all operational taxonomic units (OTUs) at 97% identity were obtained using UPARSE (version 7.0). The shared and unique OTUs between groups were visualized using Mothur software (version 1.31.2). A ranked abundance curve was drawn in R software (v3.6.1) to explain both the richness and evenness of species. Alpha diversity and richness calculations were performed using Wilcoxon rank-sum test and FDR for multiple test correction in R software (v3.6.1). UPGMA cluster analysis with bray-curtis distance matrix was performed in R software (V3.6.1). Beta diversity was compared with the representative sequences of OTUs for each group by Vegan package in R (V3.6.1) and the difference between two groups was performed using the Analysis of Similarities (ANOSIM). The Principal coordinate analysis (PCoA) display diagrams were obtained by Vegan package in R (V3.6.1), which were used to study the similarity or dissimilarity of sample community composition between samples. The difference of OTUs in all the groups were shown by Venn using R (3.6.1). The differences between groups of bacteria on phylum level and genus level was obtained by the Kruskal-Wallis H-test and performed fdr for multiple test correction and two-tailed testing in R (v3.6.1). Microbial functions were predicted using PICRUSt (version 1.0.0) and aligned to the Kyoto Encyclopedia of Genes and Genomes (KEGG) database. Differential metabolic pathways between groups were identified using the Kruskal-Wallis H-test with FDR multiple testing correction and two-tailed testing in R (v3.6.1) and obtained by heatmap figure in Graphpad Prism 10. Finally, correlation analysis between the all the genera and metabolic pathways was conducted using the Pearson coefficient and visualized with R software (Version 3.6.1, vegan package). In this study, differences were considered significant when *p* < 0.05 and extremely significant when *p* < 0.01.

## Results

### Sequencing data

After the raw data was filtered and spliced, a total of 17,634,679 high-quality clean reads were produced in all samples, with an average of 90,900 sequences per sample (ranging from 40,017 to 214,716). The average sequence length was 413 bp, with the maximum length being 418 bp and the shortest length being 408 bp.

### Microbial diversity analysis

**Figure 1:**
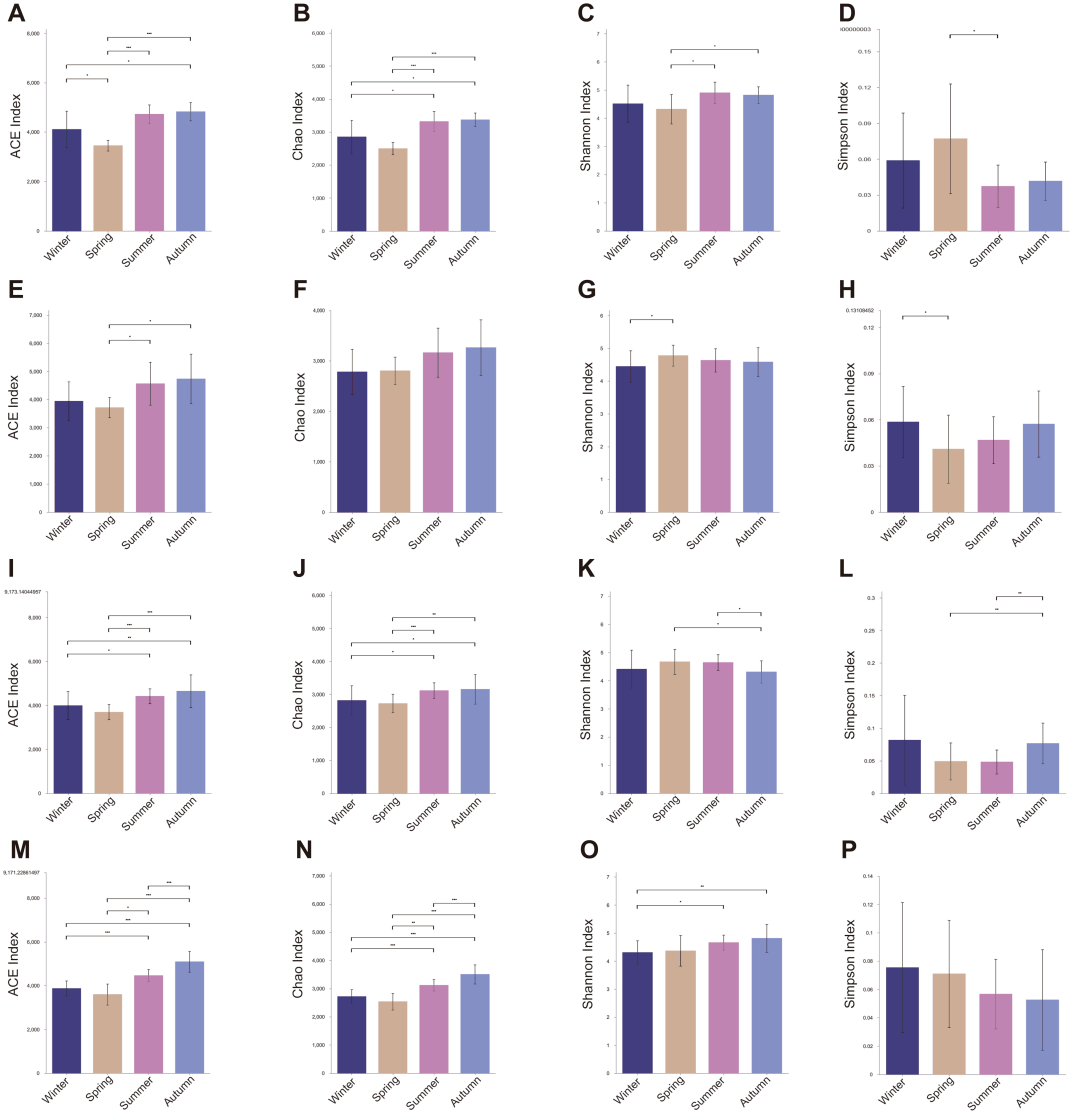
The differences in microbial diversity among all the groups in four seasons. The difference of **(A)** ACE, **(B)** Chao1, **(C)** Shannon and **(D)** Simpson index in Adult Male (AM) groups among four seasons. The difference of **(E)** ACE, **(F)** Chao1, **(G)** Shannon and **(H)** Simpson index in Subadult Male (SM) groups among four seasons. The difference of **(I)** ACE, **(J)** Chao1, **(K)** Shannon and **(L)** Simpson index in Adult Female (AF) groups among four seasons. The difference of **(M)** ACE, **(N)** Chao1, **(O)** Shannon and **(P)** Simpson index in Subadult Female groups among four seasons. (Description: *indicates significant differences, *p* < 0.05; **indicates significant differences, *p* < 0.01; ***indicates significant differences, *p* < 0.001, Wilcoxon test).

The seasonal variations in microbial diversity were primarily assessed by monitoring microbial richness (ACE index and Chao1 index) and microbial diversity (Shannon index and Simpson index). Overall, the average annual gut microbial richness was highest in SF group (ACE = 4410.58 ± 673.21, Chao = 3071.91 ± 446.99), followed by AM group (ACE = 4301.66 ± 669.23, Chao = 3019.34 ± 499.42), AF group (ACE = 4155.37 ± 680.83, Chao = 2934.54 ± 409.42), and SM group (ACE = 4081.07 ± 732.47, Chao = 2921.60 ± 450.11). Regardless of age and sex, the gut microbial diversity in golden monkeys, particularly in terms of richness indices (Figure 1), varied across the four seasons. The richness of the gut microbiota was highest in summer and autumn, followed by winter, and was lowest in spring (Figure 1). Compared to other groups, the AM group of monkeys exhibited greater fluctuations in gut microbial richness throughout the year, with significantly lower richness in spring compared to winter (ACE, *p* = 0.030; Chao, *p* = 0.079), summer (ACE, *p* < 0.001; Chao, *p* < 0.001), and autumn (ACE, *p* < 0.001; Chao, *p* < 0.001, Supplementary Table S4). Differences in gut microbial richness for the AF, SM, and SF groups were primarily concentrated between spring and summer, as well as between summer and autumn (Figure 1). Interestingly, the gut microbial diversity in the AM group of monkeys was lowest in spring (AM Group: Shannon, Spring = 4.320, Summer = 4.902, Autumn = 4.860, Winter = 4.518, Supplementary Table S3), compared to the other three groups, where diversity was lowest in winter (Figures 1D,H,L,P; Supplementary Table S3). Significant difference analysis revealed that in the AM group, gut microbial diversity in spring was significantly lower than in summer (Shannon, *p* = 0.022, Simpson, *p* = 0.037) and autumn (Shannon, *p* = 0.028, Simpson, *p* = 0.054, Figure 1; Supplementary Table S4). In contrast, the AF group exhibited significantly higher gut microbial diversity in spring (Shannon, *p* = 0.014, Simpson, *p* = 0.010) and summer (Shannon, *p* = 0.011, Simpson, *p* = 0.004) compared to autumn (Figure 1; Supplementary Table S4). In summary, adult male monkeys exhibited a significant decline in gut microbial diversity and richness in spring, whereas the changes in the other three groups were less pronounced during this season.

### Microbial community analysis

To gain insight into similarities in the bacterial community structures seasonal changes among the 4 study groups, PCoA of beta diversity analysis was performed based on the Bray-curtis distances, which demonstrated different community structures among AM, AF, SM and SF groups in different season. From an overview perspective, microbiota samples from all groups were not separated with each other, which represented higher compositional similarity in community structures with any other groups. The whole PCoA analysis with the first two principal components represented 12.64 and 6.017% of the total variations (Supplementary Figure S1). Compared to adult individuals, the gut microbiota of subadult individuals exhibited relatively stable seasonal structures, with significant differences primarily observed between spring and other seasons (Table 1). In adult males, significant seasonal changes in gut structure were mainly found between spring and other seasons, as well as between autumn and winter. In contrast, adult females showed the most pronounced changes in gut microbiota structure, with significant differences observed between any two seasons.

**Table 1:** Results of ANOSIM analyses of each group between every two seasons.

ANOSIM	R	*P*	R	*P*
	Adult male		Adult female	
Spring vs. Summer	0.502	0.001^c^	0.256	0.003^b^
Spring vs. Autumn	0.399	0.001^c^	0.666	0.001^c^
Spring vs. Winter	0.316	0.001^c^	0.561	0.001^c^
Summer vs. Autumn	0.108	0.134	0.288	0.002^b^
Summer vs. Winter	0.142	0.111	0.299	0.002 ^b^
Autumn vs. Winter	0.304	0.006^b^	0.214	0.001^c^
	Subadult male		Subadult female	
Spring vs. Summer	0.603	0.001^c^	0.349	0.011^a^
Spring vs. Autumn	0.685	0.002^b^	0.368	0.006^b^
Spring vs. Winter	0.318	0.001^c^	0.153	0.095
Summer vs. Autumn	0.091	0.163	0.078	0.077
Summer vs. Winter	0.018	0.370	0.082	0.084
Autumn vs. Winter	0.099	0.192	0.141	0.025

^a^indicates significant differences (*P* < 0.05), ^b^indicates significant differences (*P* < 0.01), ^c^indicates significant differences (*p* < 0.001).

### Microbial composition analysis

**Figure 2:**
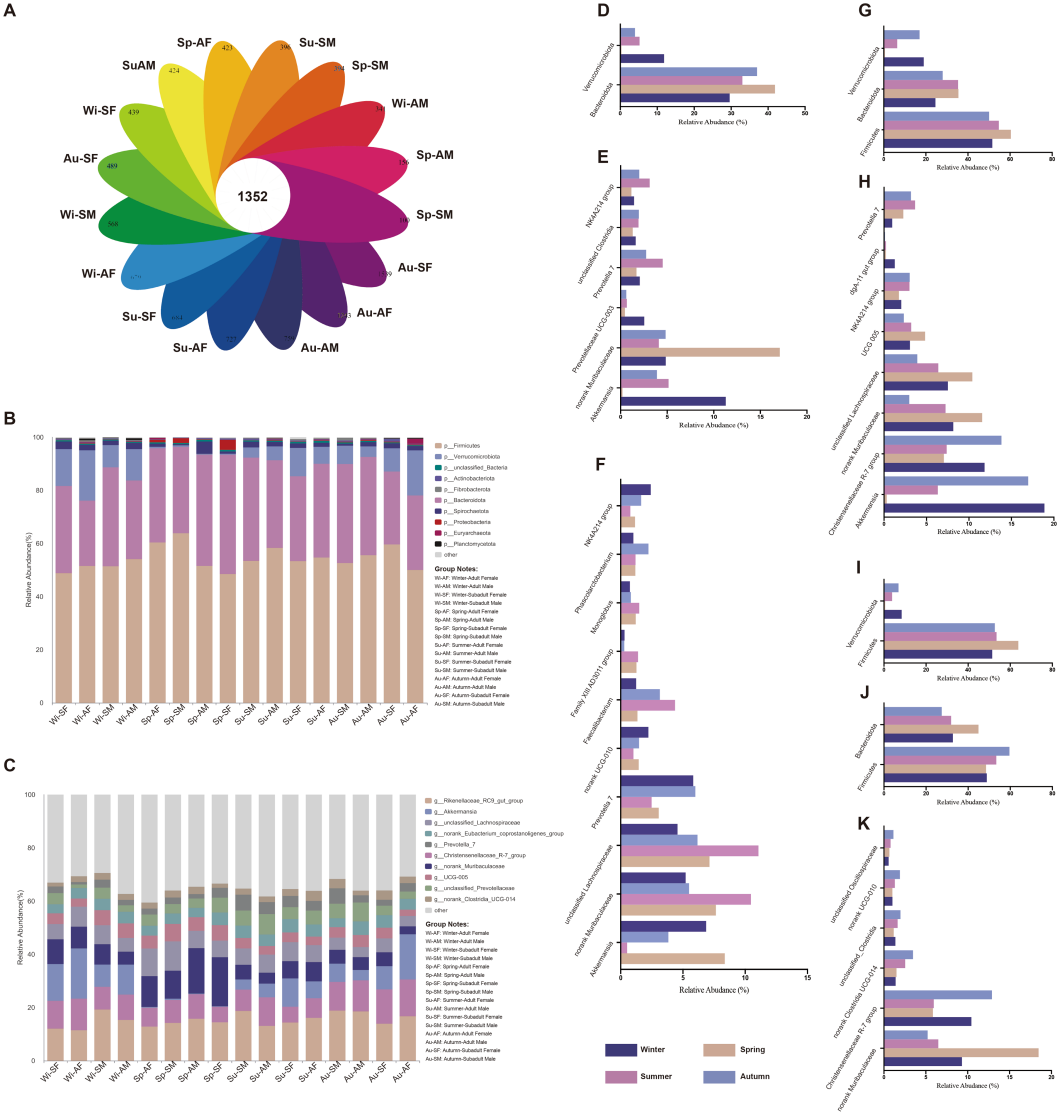
The gut microbial community and difference results. **(A)** The unique and shared OTUs among all groups. The microbial community bar plot at the **(B)** Phylum level and **(C)** Genus level. Bar charts showing the relative abundance of all phyla and genera detected in the gut microbiota collected from AM, AF, SM and SF group in different seasons. The identities of the microbiome were shown with color blocks on the right. The significant difference of gut microbial **(D)** phyla and **(E)** genera of AM samples among different seasons. The significant difference of gut microbial **(F)** genera of SM samples among different seasons. The significant difference of gut microbial **(G)** phyla and **(H)** genera of AF samples among different seasons. The significant difference of gut microbial **(I)** phyla of SM samples among different seasons. The significant difference of gut microbial **(J)** phyla and **(K)** genera of SF samples among different seasons.

The total numbers of OTUs obtained was 22,879, among which 1,352 OTUs were shared by 16 groups (four groups, each group experience four seasons), representing the core OTUs of Sichuan snub-nosed monkeys (Figure 2A). The Maximum number of unique OTUs was 1,539, which identified in the SF group during Autumn (Figure 2A), The Minimum number of unique OTUs was 100, which identified in the SF group during Spring (Figure 2A).

At the phylum level, the top three phyla in terms of relative abundance across all samples were Firmicutes, Bacteroidota, and Verrucomicrobiota, collectively accounting for approximately 99% of all phyla. From the perspective of variation across all samples, the relative abundance of Firmicutes and Bacteroidota was highest in spring (Average, Firmicutes = 56.06%, Bacteroidota = 38.69%). It gradually decreased through summer (Average, Firmicutes = 54.94%, Bacteroidota = 34.77%) and autumn (Average, Firmicutes = 54.45%, Bacteroidota = 32.42%), reaching its lowest point in winter (Average, Firmicutes = 51.47%, Bacteroidota = 31.02%). In contrast, the relative abundance of Verrucomicrobiota showed an opposite trend. It was lowest in spring (0.45%) and gradually increased through summer (6.54%) and autumn (9.17%), reaching its highest level in winter (13.28%). By the Kruskal-Wallis H-test, we found that in spring, the relative abundance of Bacteroidota significantly increased in the AM (*p* = 0.032, Figure 2D), AF (*p* = 0.006, Figure 2G) and SF groups (*p* = 0.048, Figure 2J). Similarly, Firmicutes showed a significant increase in the AF (*p* = 0.025, Figure 2G), SM (*p* = 0.014, Figure 2I), and SF groups (*p* = 0.016, Figure 2J) during spring. Conversely, phyla Verrucomicrobiota was significantly enriched in the AM (*p* = 0.011, Figure 2D), AF (*p* < 0.001, Figure 2G) and SM groups (*p* = 0.007, Figure 2I) during winter.

At genus level, the ten genera with the highest relative abundance across all samples were *Rikenellaceae RC9 gut group*, *Christensenellaceae R-7 group*, *Akkermansia*, *norank Muribaculaceae*, *unclassified Lachnospiraceae*, *UCG-005*, *norank Eubacterium coprostanoligenes group*, *unclassified Prevotellaceae*, *Prevotella7 and norank Clostridia UCG-014*, collectively accounting for approximately 60% of all genera. The differential analysis revealed that in adult male monkeys, the gut microbiota genera *norank_Muribaculaceae* (*p* < 0.001) was significantly enriched in spring, genera *Prevotella_7* (*p* = 0.048) and *NK4A214_group* (*p* < 0.001) were significantly enriched in summer, genera *unclassified_Clostridia* (*p* < 0.022) in autumn, and genera *Akkermansia* (*p* < 0.018) and *Prevotellaceae_UCG-003* (*p* < 0.005) were significantly enriched in winter (Figure 2E). In adult female monkeys, the gut microbiota genera *norank Muribaculaceae* (*p* < 0.001), u*nclassified Lachnospiraceae* (*p* < 0.001) and *UCG-005* (*p* < 0.001) were significantly enriched in spring, genera *Prevotella 7* (*p* = 0.018) was significantly enriched in summer, genera *Christensenellaceae R-7 group* (*p* < 0.001) and *NK4A214* (*p* = 0.018) group were significantly enriched in autumn, genera *Akkermansia* (*p* < 0.001) and *dgA-11 gut group* (*p* < 0.001) were significantly enriched in winter (Figure 2H). In subadult male monkeys, the gut microbiota genera *norank Muribaculaceae* (*p* = 0.003), u*nclassified Lachnospiraceae* (*p* < 0.001), *Faecalibacterium* (*p* < 0.001), Family XIII AD3011 group (*p* = 0.001) and *Monoglobus* (*p* = 0.029) were significantly enriched in spring, genera *Prevotella 7* (*p* = 0.040) and u*nclassified Phascolarctobacterium* (*p* < 0.011) were significantly enriched in summer. Genera *norank UCG-010* (*p* = 0.020) and *NK4A214 group* (*p* < 0.011) were significantly enriched in autumn, only genera *Akkermansia* (*p* = 0.007) was significantly enriched in winter (Figure 2F). In subadult female monkeys, the gut microbiota genera *norank Muribaculaceae* (*p* < 0.001) was significantly enriched in spring, genera *Christensenellaceae R-7 group* (*p* < 0.001), *norank Clostridia UCG-014* (*p* = 0.029), *unclassified Clostridia* (*p* < 0.011), *norank UCG-010* (*p* = 0.003) and *unclassified Oscillospiraceae* (*p* < 0.001) were significantly enriched in autumn (Figure 2K).

### Predictive function gene analysis

Across all samples in the four groups, we identified a total of 38 KEGG level 2 functional metabolic pathways, which belong to 6 KEGG level 1 functional categories. The metabolic pathway with the highest relative abundance was Metabolism, accounting for approximately 68.91% (Supplementary Figure S2). The remaining five pathways, in descending order of relative abundance, were Genetic Information Processing (13.43%), Environmental Information Processing (6.91%), Cellular Community (5.01%), Human Diseases (3.70%), and Organismal Systems (2.03%, Supplementary Figure S2).

**Figure 3:**
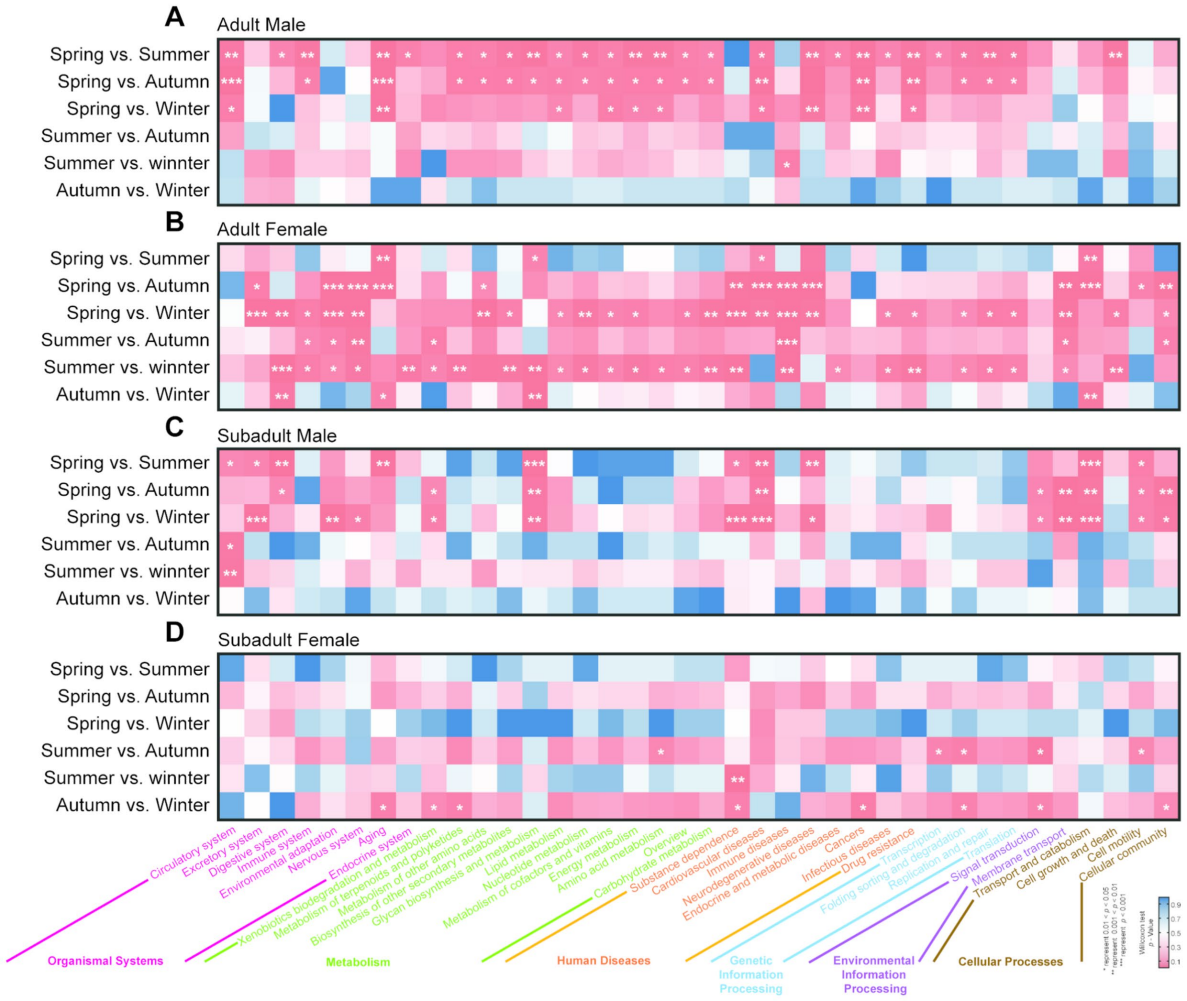
Seasonal variation significance analysis of KEGG level 2 functional metabolic pathways in the gut microbiota of golden snub-nosed monkeys. The figures showed the seasonal significance analysis of KEGG level 2 functional metabolic pathways for **(A)** AM group, **(B)** AF group, **(C)** SM group, and **(D)** SF group. The x-axis represents the names of the corresponding KEGG level 2 and level 1 metabolic pathways. Functional pathways for different groups are distinguished by different colors. The significance of differences is indicated by a blue-to-red gradient, with * denoting a significance level of *p* < 0.05, ** for *p* < 0.01, and *** for *p* < 0.001.

Through the seasonal difference analysis of gut microbiota metabolic functions for each group, we identified 58 significant differences in the AM group across the four seasons. Notably, more than 98% of these differences occurred between spring and the other three seasons (summer, autumn, and winter, Figure 3). The primary impacted functions were within the metabolic functional pathways, with 26 significant occurrences. In spring, 12 metabolic pathways, including Carbohydrate Metabolism (spring vs. summer, *p* = 0.031; spring vs. autumn, *p* = 0.028), Amino Acid Metabolism (spring vs. summer, *p* = 0.008; spring vs. autumn, *p* = 0.017), Energy Metabolism (spring vs. summer, *p* = 0.008; spring vs. autumn, *p* = 0.017), and Lipid Metabolism (spring vs. summer, *p* = 0.031; spring vs. autumn, *p* = 0.022), were significantly lower compared to summer and autumn (Figure 3A). Similarly, in the seasonal analysis of gut microbiota in the AF group, we identified 80 functional differences. Unlike the AM group, the AF group exhibited differences primarily concentrated between winter and spring, as well as winter and summer, with 56 occurrences, accounting for approximately 70% of all differential metabolic pathways. Compared to spring and summer, 22 metabolic-related pathways such as Carbohydrate Metabolism (spring vs. winter, *p* = 0.007; summer vs. winter, *p* = 0.010), Energy Metabolism (spring vs. winter, *p* = 0.026; summer vs. winter, *p* = 0.011), Nucleotide Metabolism (spring vs. winter, *p* = 0.007; summer vs. winter, *p* = 0.010), and Lipid Metabolism (spring vs. winter, *p* = 0.009; summer vs. winter, *p* = 0.012) significantly decreased in winter (Figure 3B).

Unlike adult individuals, the gut microbiota functional metabolic pathways in subadult individuals are less affected by seasonal changes. For instance, in the SM group, there were 34 significant differences in gut microbiota functional metabolic pathways when comparing pairs of seasons. These differences were primarily concentrated between spring and the other three seasons (summer, autumn, and winter). Notably, pathways related to Cellular Community and Environmental Information Processing, such as Cellular Community (spring vs. summer, *p* = 0.149; spring vs. autumn, *p* = 0.006; spring vs. winter, *p* = 0.011), Cell Motility (spring vs. summer, *p* = 0.016; spring vs. autumn, *p* = 0.029; spring vs. winter, *p* = 0.030), Membrane Transport (spring vs. summer, *p* = 0.190; spring vs. autumn, *p* = 0.002; spring vs. winter, *p* = 0.001), and Signal Transduction (spring vs. summer, *p* = 0.056; spring vs. autumn, *p* = 0.036; spring vs. winter, *p* = 0.041), were significantly enriched in spring. Compared to the other three groups, the gut microbiota functional metabolic pathways in the SF group were the least affected by seasonal changes, with only 14 significant differences observed. Among these, only three were related to metabolic functional pathways. For example, in the SF group, the Amino Acid Metabolism pathway in autumn was significantly higher than in summer (*p* = 0.043), while the pathways for Metabolism of Terpenoids and Polyketides (*p* = 0.028) and Xenobiotics Biodegradation and Metabolism (*p* = 0.049) in autumn were significantly higher than in winter.

### The correlation analysis between functional metabolic pathways and microbial genera

**Figure 4:**
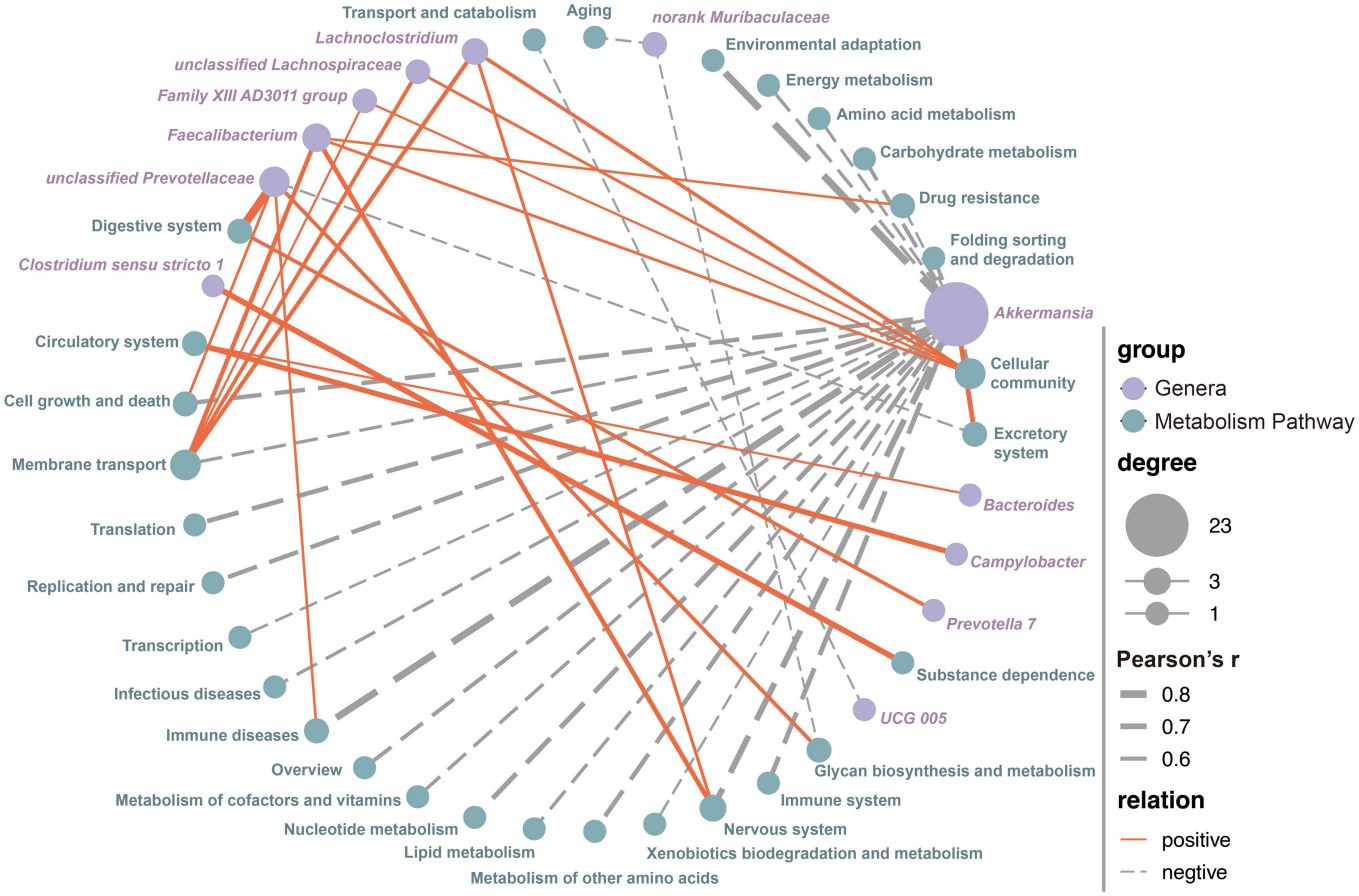
The correlation between all the microbial genera and metabolism pathways. The correlations were using the Spearman method and only the robust and significant correlation (correlation values < −0.5 or >0.5 and *p* < 0.05) were kept for the construction of co-occurrence networks. Potential keystone taxa on the basis of co-occurrence network analyses of microbial communities and metabolism pathways, node sizes and colors are proportional to their scaled NESH score (i.e., degree revealing important microbial taxa of microbial association networks). The size of the dots indicates the importance of each genera or metabolism pathway, while the thickness of the connecting lines reflects the strength of the correlation. Red lines represent positive correlations, and gray dotted lines indicate negative correlations.

The Pearson correlation analysis was performed to explore the potential relationships between all the microbial genera and metabolic pathways. By establishing stringent criteria (*r* > 0.5 or *r* < −0.5, and *p* < 0.05), we identified 46 potential correlations between bacterial genera and metabolic pathways. These included 12 genera and 29 KEGG level 2 metabolic pathways. Among these potential relationships, 22 metabolic pathways, including Amino Acid Metabolism (*r* = −0.567, *p* = 4.232E-18), Carbohydrate Metabolism (*r* = −0.614, *p* = 1.083E-21), Energy Metabolism (*r* = −0.582, *p* = 3.790E-19), and Lipid Metabolism (*r* = −0.589, *p* = 1.017E-19), showed a significant negative correlation with the genera *Akkermansia*, while only the Excretory System was positively correlated (*r* = 0.661, *p* = 5.303E-26, Figure 4; Supplementary Table S5). Notably, almost all metabolism-related pathways were significantly associated with the relative abundance of *Akkermansia* (Figure 4; Supplementary Table S5). Additionally, an increase in the relative abundance of *unclassified Prevotellaceae* significantly enhanced the expression of functions related to the Digestive System (*r* = 0.850, *p* = 7.26E-56), Glycan Biosynthesis and Metabolism (*r* = 0.607, *p* = 4.234E-21), Cell Growth and Death (*r* = 0.554, *p* = 3.626E-17), and Immune Diseases (*r* = 0.546, *p* = 1.284E-16), while reducing the expression of the Excretory System (*r* = −0.532, *p* = 9.692E-16, Figure 4; Supplementary Table S5). An increase in the relative abundance of *Faecalibacterium* significantly enhanced the expression of functions related to Cellular Community (*r* = 0.559, *p* = 1.745E-17), Drug Resistance (*r* = 0.543, *p* = 2.085E-16), Membrane Transport (*r* = 0.638, *p* = 7.931E-24), and the Nervous System (*r* = 0.655, *p* = 2.036E-25, Figure 4; Supplementary Table S5). Similarly, an increase in the relative abundance of *Lachnoclostridium* significantly enhanced the expression of functions related to Cellular Community (*r* = 0.600, *p* = 1.622E-20), Membrane Transport (*r* = 0.628, *p* = 6.721E-23), and the Nervous System (*r* = 0.576, *p* = 1.057E-18). The remaining eight genera were correlated with only one to two metabolic pathways.

## Discussion

According to the article “Observational Study of Behavior: Sampling Methods,” there are various behavioral sampling methods for animal observational studies (Altmann, 1974). Based on these observational research methods, previous researchers have studied behaviors such as grooming (Zhang et al., 2010), mutual hugging (Zhao et al., 2015), conflict and reconciliation (Zhao et al., 2015), social play (Qi et al., 2009), infanticide (Xiang et al., 2022), and alloparenting (Ren et al., 2012; Xiang et al., 2019) in golden monkeys. Observational research methods demand significant time and involvement from observers (Peregrine et al., 1993; Sharp et al., 2015). Previous studies on the gut microbiota of Sichuan snub-nosed monkeys have generally involved sizes of fewer than 50, with <50% of the samples being fresh and collected from the wild. This limitation hinders the characterization of gut microbiota variations wild populations of these monkeys (Zhu et al., 2018; Wang et al., 2023; Xi et al., 2023; Zhao et al., 2023). In this study, we innovatively utilized high-throughput sequencing of gut microbiota from 194 fecal samples to explore how wild adult male golden monkeys ensure the healthy development of their families under conditions of food scarcity. It is the first to demonstrate the importance of altruistic behavior expression by adult male monkeys in the spring. Additionally, it provides preliminary research data on the potential role of microbiota in influencing the behavior of golden monkeys.

Like other studies, our research found that the gut microbiota diversity of adult male Sichuan snub-nosed monkeys significantly decreases in the spring (Wang et al., 2023). Both the ACE and Chao indices were notably lower in the spring compared to other seasons, with significant differences observed when compared to winter, summer, and autumn. Spring is a critical period for rearing young and is also a time of relative food scarcity (Xiang et al., 2017; Tuyisingize et al., 2023). Previous studies have demonstrated that a monotonous diet and scarcity of food resources can directly disrupt the stability of microbial community structures, leading to a decline in diversity (Overbeeke et al., 2022). For instance, Sichuan snub-nosed monkeys (*Rhinopithecus roxellana*) (Liu et al., 2024), Orangutans (*Pongo abelii*) (Bo et al., 2023), Rhesus macaques (*Macaca mulatta*) (Sun et al., 2021), and Chimpanzees (*Pan troglodytes*) (Quiroga-González et al., 2021) transferred to captive environments experience a reduction in gut microbiota diversity due to the limited variety in their diet. Additionally, one major factor inducing changes in gut microbiota diversity and composition is the alteration of environmental temperature. Variations in temperature may indirectly affect gut microbial communities by influencing the host’s metabolic rate, dietary habits, and energy intake (Zhu et al., 2021; Liu et al., 2023; Shuai He et al., 2024). Here, we speculate that another reason causing this decline in microbial diversity during the spring may be closely related to altruistic behavior. Adult males may reduce their own food intake to allocate more resources to pregnant and nursing females, ensuring the survival of their offspring (de Waal, 1997; Nettelbeck, 1998). This change in food resource allocation could affect gut microbiota diversity, as reduced food intake may alter the nutrients entering the gut, impacting the growth and reproduction environment of the microbiota (Erkosar et al., 2018; Sbierski-Kind et al., 2022). Correspondingly, the gut bacterial genera of adult males also changed, with *norank Muribaculaceae* significantly enriched in the spring, whereas *Akkermansia* and *Prevotellaceae UCG-003* were significantly enriched in the winter. *Akkermansia* has been shown to regulate the host’s basal metabolism by influencing energy metabolism and the immune system, thereby enhancing the efficiency of carbohydrate and fat breakdown (Wu et al., 2022; Wu et al., 2023). *Muribaculaceae* can produce short-chain fatty acids (SCFAs) from both endogenous and exogenous polysaccharides, such as dietary fibers (Acharya and Bajaj, 2020). Additionally, it forms cross-feeding relationships with other beneficial bacteria like *Bifidobacteria* and *Lactobacilli*, which help nourish the host (Zhu et al., 2024). During winter, adult males may need to maintain better physical condition to cope with the cold environment and possibly to fulfill protective or competitive roles within the group, such as defending the group or competing for mates (Huang et al., 2017). An increase in the relative abundance of *Akkermansia* may help regulate metabolism and improve the efficiency of resource utilization, aligning with altruistic behavior by supporting the group while meeting basic survival needs. The enrichment of *norank Muribaculaceae* in spring may relate to the characteristics of food resources and the unique physiological and behavioral states of males during this period (low-carbohydrate or high-fiber diets, such as bark), potentially participating in the metabolism of specific food components to adapt to changes in food resource allocation (Acharya and Bajaj, 2020). In this study, the transition in adult male Sichuan snub-nosed monkeys from a winter gut type enriched with *Akkermansia* to a spring gut type enriched with *Muribaculaceae*, along with changes in microbiota diversity, mutually reinforce the expression of altruistic behavior.

Changes in metabolic pathways also reflect a correlation with altruistic behavior. In spring, various metabolic pathways of the gut microbiota in adult males, such as carbohydrate, amino acid, energy, and lipid metabolism, were significantly lower than in summer and autumn. This aligns with the situation where food resources are allocated to females for rearing young (Luo et al., 2011). Reduced food intake leads to fewer substrates available for metabolism by the gut microbiota, resulting in decreased activity in these metabolic pathways (Rowland, 1988). This metabolic adjustment may be an adaptive change by adult males to prioritize the nutritional needs of nursing females and offspring when resources are limited. In the long term, these adaptive changes in metabolic pathways contribute to the survival and reproduction of the entire group, representing a physiological manifestation of altruistic behavior.

Furthermore, the altruistic behavior of adult males in sharing food with related females and juveniles during periods of food scarcity can enhance their leadership charisma and reliability (Wooders and Berg, 2001; Dreher et al., 2016). For example, in wild Guinea baboons, males share meat with females in their reproductive units after a successful hunt (Goffe and Fischer, 2016). This sharing behavior may help males maintain female loyalty, while females gain access to rare, nutrient-rich food sources (Goffe and Fischer, 2016). In the society of Sichuan snub-nosed monkeys, female transfer is one of the primary ways new one-male units (OMUs) are formed, often involving two or more females moving together to a new OMU or group (Qi et al., 2009; Guo et al., 2015). During the formation of these groups, fights between adult males are rarely observed, and female mate choice appears to play a crucial role in the recruitment and replacement of group leaders (Qi et al., 2009). This suggests that the social structure of Sichuan snub-nosed monkeys heavily relies on female choice behavior. Thus, when adult male Sichuan snub-nosed monkeys relinquish food to females for childbirth and rearing during the resource-scarce spring, it not only improves the survival and health of the offspring but also consolidates unit population development by appeasing members and establishing prestige, thereby promoting the dissemination of their genes.

Overall, the diversity of gut microbiota, bacterial genera, and metabolic pathways in adult male Sichuan snub-nosed monkeys interact and influence each other, forming a complex network associated with altruistic behavior. Seasonal changes in microbial diversity affect the relative abundance of bacterial genera, which in turn regulate the activity of metabolic pathways (David et al., 2014). In spring, lower microbial diversity and the enrichment of specific bacterial genera may collectively drive adaptive changes in metabolic pathways, reducing the males’ demand for food resources and allocating more resources to nursing females and offspring, thereby exhibiting altruistic behavior. This altruistic behavior not only benefits the survival and growth of the young within the group, enhancing the overall adaptability of the group, but may also, through feedback mechanisms within the group, influence the gut microbiota of adult males (Moeller et al., 2016; Johnson et al., 2022). In other seasons, group sharing of food resources may help restore and adjust their gut microbiota, maintaining individual health and group stability. This series of correlations reveals the important role of gut microbiota in regulating social behavior in animals, providing new insights into the behavioral evolution and group ecology of animals like the Sichuan snub-nosed monkey. The sequencing depth in this study may have limited the functions and pathways identified in the final analysis. Future studies could use more accurate sequencing methods, such as metagenomes.

## Data Availability

The raw reads were deposited into the NCBI Sequence Read Archive (SRA) database (BioProject: PRJNA1172943).
