# Supplementary material for: Gut microbiota alterations in golden snub-nosed monkeys during food shortage and parturition-nursing periods
Source: Front Microbiol. 2025 Feb 27;16:1556648. doi: 10.3389/fmicb.2025.1556648 (PMC11903488; doi:10.3389/fmicb.2025.1556648)
Supplement: Supplementary file 1 [file Table_1.doc]

**Gut Microbiota Alterations in Golden Snub-Nosed Monkeys During Food Shortage and Parturition-Nursing Periods**

**Table S1. Seasonal variation in the diet of Sichuan snub-nosed monkeys**

| Food | Season | | | |
| --- | --- | --- | --- | --- |
|  | Winter^a^ | Spring^abc^ | Summer^c^ | Autumn |
| Mature leaves | 3.6% | 0.8% | 44.8% | 29.9% |
| Young leaves | 2.1% | 32.6% | 25.6% | 24.2% |
| Fruits or seeds | 4.2% | 0.6% | 2.7% | 35.6% |
| Bark | 48.6% | 32.5% | 4.8% | 0.5% |
| Others | 41.5% | 33.5% | 22.1% | 0.9% |

^a^ indicates food shortage period. ^b^ indicates parturition period. ^c^ indicates nursing period.
