# Supplementary material for: Gut microbiota alterations in golden snub-nosed monkeys during food shortage and parturition-nursing periods
Source: Front Microbiol. 2025 Feb 27;16:1556648. doi: 10.3389/fmicb.2025.1556648 (PMC11903488; doi:10.3389/fmicb.2025.1556648)
Supplement: Supplementary file 2 [file Table_2.doc]

**Gut Microbiota Alterations in Golden Snub-Nosed Monkeys During Food Shortage and Parturition-Nursing Periods**

**Table S2. The information of all the samples**

| Monkeys of different  ages and genders | Season | | | |
| --- | --- | --- | --- | --- |
|  | Spring^abc^ | Summer^c^ | Autumn | Winter^a^ |
| Adult-Male | (Sp-AM)  n = 9 | (Su-AM)  n = 7 | (Au-AM)  n = 10 | (Wi-AM)  n = 9 |
| Adult-Female | (Sp-AF)  n = 19 | (Su-AF)  n = 13 | (Au-AF)  n = 16 | (Wi-AF)  n = 18 |
| Subadult-Male | (Sp-SM)  n = 15 | (Su-SM)  n = 8 | (Au-SM)  n = 6 | (Wi-SM)  n = 16 |
| Subadult-Female | (Sp-SF)  n = 5 | (Su-SF)  n = 14 | (Au-SF)  n = 16 | (Wi-SF)  n = 13 |

^a^ indicates food shortage period. ^b^ indicates parturition period. ^c^ indicates nursing period.
