# Supplementary material for: Gut microbiota alterations in golden snub-nosed monkeys during food shortage and parturition-nursing periods
Source: Front Microbiol. 2025 Feb 27;16:1556648. doi: 10.3389/fmicb.2025.1556648 (PMC11903488; doi:10.3389/fmicb.2025.1556648)
Supplement: Supplementary file 3 [file Table_3.doc]

**Gut Microbiota Alterations in Golden Snub-Nosed Monkeys During Food Shortage and Parturition-Nursing Periods**

**Table S3. The gut microbial diversity of all the samples among four seasons**

|  | Spring | | Summer | | Autumn | | Winter | |
| --- | --- | --- | --- | --- | --- | --- | --- | --- |
|  | Mean | SD | Mean | SD | Mean | SD | Mean | SD |
| Adult-Male | | | | | | | | |
| ace | 3452.740 | 217.559 | 4724.787 | 377.643 | 4942.940 | 509.010 | 4108.929 | 738.765 |
| chao | 2500.226 | 186.853 | 3317.863 | 303.592 | 3429.193 | 262.672 | 2850.884 | 505.288 |
| shannon | 4.320 | 0.523 | 4.902 | 0.380 | 4.860 | 0.310 | 4.518 | 0.660 |
| simpson | 0.077 | 0.046 | 0.037 | 0.018 | 0.040 | 0.016 | 0.059 | 0.040 |
| Subadult-Male | | | | | | | | |
| ace | 3713.548 | 360.566 | 4561.896 | 760.311 | 4732.008 | 881.075 | 3941.119 | 689.313 |
| chao | 2803.863 | 271.792 | 3164.504 | 487.316 | 3262.486 | 553.112 | 2782.683 | 447.371 |
| shannon | 4.779 | 0.320 | 4.635 | 0.356 | 4.584 | 0.438 | 4.449 | 0.479 |
| simpson | 0.041 | 0.022 | 0.047 | 0.015 | 0.057 | 0.021 | 0.059 | 0.023 |
| Adult-Female | | | | | | | | |
| ace | 3693.042 | 347.610 | 4414.329 | 338.031 | 4645.476 | 744.301 | 3993.480 | 638.191 |
| chao | 2724.423 | 280.762 | 3115.289 | 237.470 | 3156.124 | 449.398 | 2816.521 | 446.378 |
| shannon | 4.670 | 0.441 | 4.644 | 0.279 | 4.309 | 0.395 | 4.410 | 0.674 |
| simpson | 0.049 | 0.028 | 0.048 | 0.018 | 0.077 | 0.031 | 0.082 | 0.069 |
| Subadult-Female | | | | | | | | |
| ace | 3595.445 | 481.272 | 4460.161 | 277.284 | 5092.926 | 477.807 | 3872.311 | 344.317 |
| chao | 2536.046 | 293.137 | 3119.920 | 204.491 | 3505.145 | 338.524 | 2720.166 | 245.081 |
| shannon | 4.367 | 0.546 | 4.661 | 0.270 | 4.812 | 0.496 | 4.308 | 0.418 |
| simpson | 0.071 | 0.038 | 0.057 | 0.025 | 0.053 | 0.035 | 0.075 | 0.046 |
