# Supplementary material for: Gut microbiota alterations in golden snub-nosed monkeys during food shortage and parturition-nursing periods
Source: Front Microbiol. 2025 Feb 27;16:1556648. doi: 10.3389/fmicb.2025.1556648 (PMC11903488; doi:10.3389/fmicb.2025.1556648)
Supplement: Supplementary file 4 [file Table_4.doc]

**Gut Microbiota Alterations in Golden Snub-Nosed Monkeys During Food Shortage and Parturition-Nursing Periods**

Table S4. The result in significant analysis of seasonal gut microbial diversity

| Willcoxon rank-sum test | | | | | | |
| --- | --- | --- | --- | --- | --- | --- |
|  | *P* (Spring vs. Summer) | *P* (Spring vs. Autumn) | *P* (Spring vs. Winnter) | *P* (Summer vs. Autumn) | *P* (Summer vs. Winter) | *P* (Autumn vs. Winter) |
| Adult-Male | | | | | | |
| ace | 0.000^c^ | 0.000^c^ | 0.030^a^ | 0.601 | 0.051 | 0.023 |
| chao | 0.000^c^ | 0.000^c^ | 0.079 | 0.689 | 0.039^a^ | 0.016^a^ |
| shannon | 0.022^a^ | 0.028^a^ | 0.492 | 0.643 | 0.167 | 0.238 |
| simpson | 0.037^a^ | 0.054 | 0.383 | 0.623 | 0.173 | 0.253 |
| Subadult-Male | | | | | | |
| ace | 0.016^a^ | 0.036^a^ | 0.257 | 0.713 | 0.074 | 0.085 |
| chao | 0.083 | 0.101 | 0.874 | 0.737 | 0.086 | 0.095 |
| shannon | 0.355 | 0.354 | 0.031^a^ | 0.822 | 0.298 | 0.544 |
| simpson | 0.467 | 0.152 | 0.038^a^ | 0.336 | 0.150 | 0.901 |
| Adult-Female | | | | | | |
| ace | 0.000 ^c^ | 0.000 ^c^ | 0.089 | 0.267 | 0.025^a^ | 0.009^b^ |
| chao | 0.000 ^c^ | 0.002^b^ | 0.461 | 0.751 | 0.023^a^ | 0.032^a^ |
| shannon | 0.843 | 0.014^a^ | 0.179 | 0.011^a^ | 0.198 | 0.590 |
| simpson | 0.915 | 0.010^a^ | 0.076 | 0.004^b^ | 0.063 | 0.780 |
| Subadult-Female | | | | | | |
| ace | 0.013^a^ | 0.001^b^ | 0.285 | 0.000 ^c^ | 0.000^c^ | 0.000^c^ |
| chao | 0.008^b^ | 0.000 ^c^ | 0.255 | 0.001^b^ | 0.000^c^ | 0.000^c^ |
| shannon | 0.304 | 0.154 | 0.833 | 0.301 | 0.014^a^ | 0.005^b^ |
| simpson | 0.466 | 0.371 | 0.837 | 0.712 | 0.194 | 0.144 |

^a^ indicates significant differences (p < 0.05), ^b^ indicates significant differences (p < 0.01), ^c^ indicates significant differences (p < 0.001).
