# Supplementary material for: Gut microbiota alterations in golden snub-nosed monkeys during food shortage and parturition-nursing periods
Source: Front Microbiol. 2025 Feb 27;16:1556648. doi: 10.3389/fmicb.2025.1556648 (PMC11903488; doi:10.3389/fmicb.2025.1556648)
Supplement: Supplementary file 5 [file Table_5.doc]

**Gut Microbiota Alterations in Golden Snub-Nosed Monkeys During Food Shortage and Parturition-Nursing Periods**

**Table S5. Results of correlation analysis between functional Metabolic Pathways and microbial genera**

| Genera | Metabolism pathways | Person r | *P*-value |
| --- | --- | --- | --- |
| *Akkermansia* | Amino acid metabolism | -0.567 | 4.23E-18 |
| *Akkermansia* | Carbohydrate metabolism | -0.614 | 1.08E-21 |
| *Akkermansia* | Cellular community | -0.596 | 3.27E-20 |
| *Akkermansia* | Drug resistance | -0.539 | 3.44E-16 |
| *Akkermansia* | Energy metabolism | -0.582 | 3.79E-19 |
| *Akkermansia* | Environmental adaptation | -0.783 | 6.82E-42 |
| *Akkermansia* | Excretory system | 0.661 | 5.30E-26 |
| *Akkermansia* | Folding sorting and degradation | -0.604 | 7.82E-21 |
| *Akkermansia* | Cell growth and death | -0.639 | 6.69E-24 |
| *Akkermansia* | Infectious diseases | -0.583 | 2.87E-19 |
| *Akkermansia* | Immune diseases | -0.773 | 3.67E-40 |
| *Akkermansia* | Immune system | -0.660 | 7.02E-26 |
| *Akkermansia* | Lipid metabolism | -0.589 | 1.02E-19 |
| *Akkermansia* | Metabolism of cofactors and vitamins | -0.592 | 6.77E-20 |
| *Akkermansia* | Metabolism of other amino acids | -0.644 | 2.16E-24 |
| *Akkermansia* | Membrane transport | -0.580 | 4.85E-19 |
| *Akkermansia* | Nervous system | -0.718 | 2.21E-32 |
| *Akkermansia* | Nucleotide metabolism | -0.667 | 1.49E-26 |
| *Akkermansia* | Overview | -0.617 | 5.75E-22 |
| *Akkermansia* | Replication and repair | -0.639 | 6.42E-24 |
| *Akkermansia* | Transcription | -0.551 | 5.63E-17 |
| *Akkermansia* | Translation | -0.654 | 2.61E-25 |
| *Akkermansia* | Xenobiotics biodegradation and metabolism | -0.538 | 3.93E-16 |
| *Bacteroides* | Circulatory system | 0.527 | 2.11E-15 |
| *Campylobacter* | Circulatory system | 0.693 | 1.97E-29 |
| *Clostridium sensu stricto 1* | Substance dependence | 0.709 | 3.38E-31 |
| *Faecalibacterium* | Cellular community | 0.559 | 1.74E-17 |
| *Faecalibacterium* | Drug resistance | 0.543 | 2.08E-16 |
| *Faecalibacterium* | Membrane transport | 0.638 | 7.93E-24 |
| *Faecalibacterium* | Nervous system | 0.655 | 2.04E-25 |
| *Family XIII AD3011 group* | Cellular community | 0.532 | 9.63E-16 |
| *Family XIII AD3012 group* | Membrane transport | 0.528 | 1.95E-15 |
| *Lachnoclostridium* | Cellular community | 0.600 | 1.62E-20 |
| *Lachnoclostridium* | Membrane transport | 0.628 | 6.72E-23 |
| *Lachnoclostridium* | Nervous system | 0.576 | 1.06E-18 |
| *norank Muribaculaceae* | Aging | -0.533 | 9.41E-16 |
| *norank Muribaculaceae* | Glycan biosynthesis and metabolism | -0.520 | 5.94E-15 |
| *Prevotella 7* | Digestive system | 0.603 | 9.35E-21 |
| *UCG 005* | Transport and catabolism | -0.526 | 2.48E-15 |
| *unclassified Lachnospiraceae* | Cellular community | 0.563 | 9.13E-18 |
| *unclassified Lachnospiraceae* | Membrane transport | 0.607 | 3.89E-21 |
| *unclassified Prevotellaceae* | Digestive system | 0.850 | 7.26E-56 |
| *unclassified Prevotellaceae* | Excretory system | -0.532 | 9.69E-16 |
| *unclassified Prevotellaceae* | Glycan biosynthesis and metabolism | 0.607 | 4.23E-21 |
| *unclassified Prevotellaceae* | Cell growth and death | 0.554 | 3.63E-17 |
| *unclassified Prevotellaceae* | Immune diseases | 0.546 | 1.28E-16 |
