# Supplementary material for: Gut microbiota alterations in golden snub-nosed monkeys during food shortage and parturition-nursing periods
Source: Front Microbiol. 2025 Feb 27;16:1556648. doi: 10.3389/fmicb.2025.1556648 (PMC11903488; doi:10.3389/fmicb.2025.1556648)
Supplement: Supplementary file 6 [file Data_Sheet_1.doc]

**Gut Microbiota Alterations in Golden Snub-Nosed Monkeys During Food Shortage and Parturition-Nursing Periods**


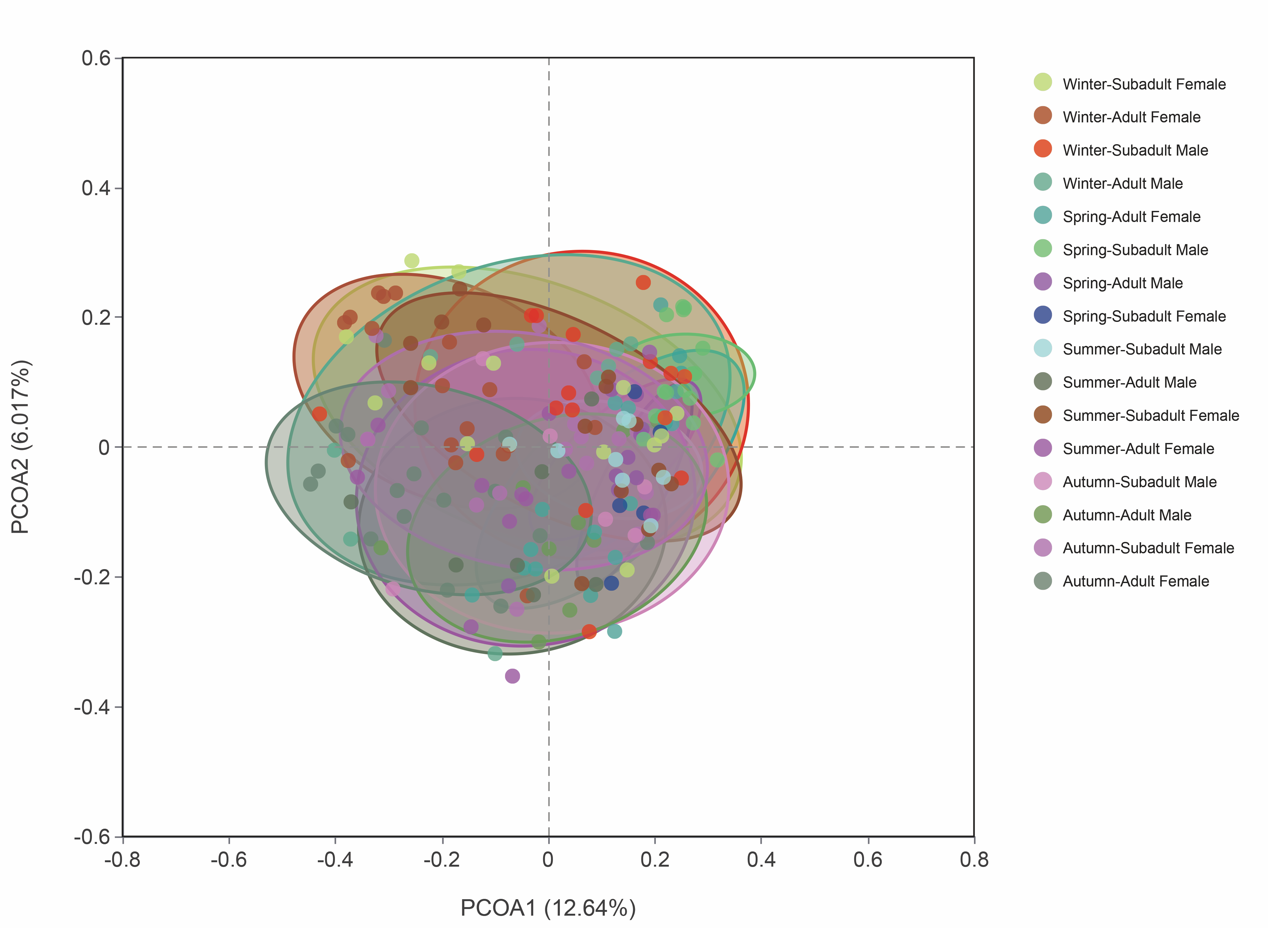


**Figure S1.** The PCoA analysis of seasonal gut microbial community among groups. PCoA was based on the Bray-curtis distance matrix to find principal coordinates. (Description: X-axis, PCoA axis1 and Y axis, PCoA axis2. The scale of the X-axis and the Y-axis are the projection coordinates of the sample points in the two-dimensional plane, respectively. A dot represents each sample, and different colors represent different groups).
