# Supplementary material for: Gut microbiota alterations in golden snub-nosed monkeys during food shortage and parturition-nursing periods
Source: Front Microbiol. 2025 Feb 27;16:1556648. doi: 10.3389/fmicb.2025.1556648 (PMC11903488; doi:10.3389/fmicb.2025.1556648)
Supplement: Supplementary file 7 [file Data_Sheet_2.doc]

**Gut Microbiota Alterations in Golden Snub-Nosed Monkeys During Food Shortage and Parturition-Nursing Periods**


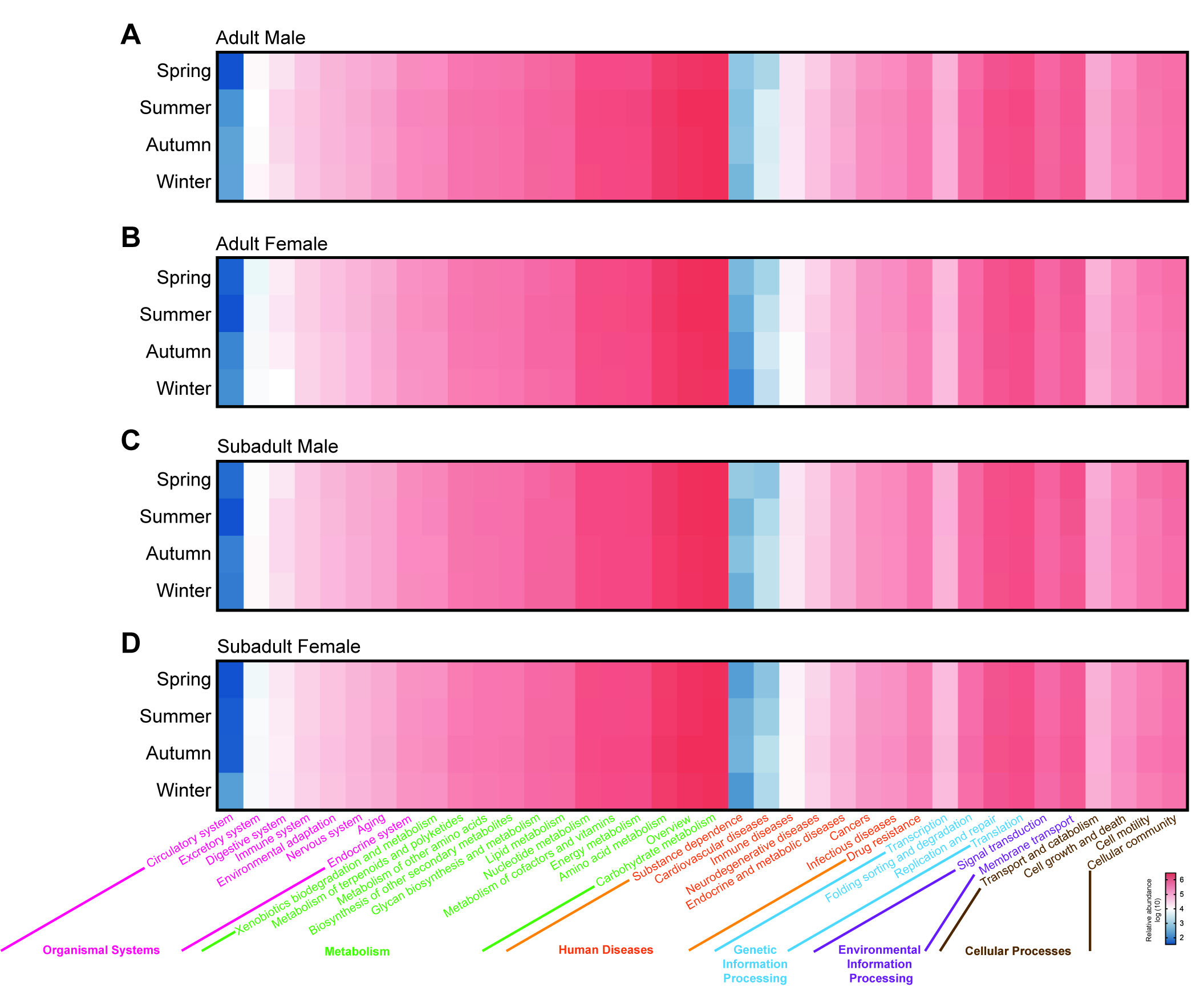


**Figure S2.** Functional predictions of the gut microbiota of golden snub-nosed monkeys at KEGG level 2. The figures show relative abundance of KEGG level 2 functions for (A) AM group, (B) AF group, (C) SM group, and (D) SF group. The x-axis represents the names of the corresponding KEGG level 2 and level 1 metabolic pathways. Functional pathways for different groups are distinguished by different colors. All data in the figure are presented after log(10) transformation, with relative abundance displayed from low to high using a blue-to-red gradient.
